# Dual Role of *Cutibacterium acnes*: Commensal Bacterium and Pathogen in Ocular Diseases

**DOI:** 10.3390/microorganisms12081649

**Published:** 2024-08-12

**Authors:** Tomo Suzuki, Shigeru Kinoshita

**Affiliations:** 1 Department of Ophthalmology, Kyoto Prefectural University of Medicine, Kyoto 602-0841, Japan; 2 Department of Ophthalmology, Kyoto City Hospital Organization, Kyoto 604-8845, Japan; 3 Department of Frontier Medical Science and Technology for Ophthalmology, Kyoto Prefectural University of Medicine, Kyoto 602-0841, Japan; shigeruk@koto.kpu-m.ac.jp

**Keywords:** *Cutibacterium acnes*, commensal bacterium, microbiome, granuloma, meibomian gland, meibomitis, keratitis, meibomitis-related keratoconjunctivitis (MRKC), ocular surface, chalazion, ocular sarcoidosis, late-onset endophthalmitis

## Abstract

Microbiota present around the ocular surface, encompassing the eyelid skin, the conjunctival sac, and the meibomian glands, play a significant role in various inflammatory conditions associated with the ocular surface. *Cutibacterium acnes* (*C. acnes*), formerly, *Propionibacterium acnes*, is one of the most predominant commensal bacteria and its relative abundance declines with aging. However, it can act as both an infectious and an immunogenic pathogen. As an infectious pathogen, *C. acnes* has been reported to cause late onset endophthalmitis post-cataract surgery and infectious keratitis. On the other hand, it can trigger immune responses resulting in conditions such as phlyctenules in the cornea, chalazion in the meibomian glands, and granuloma formation in ocular sarcoidosis. This review explores the role of *C. acnes* in ocular inflammation, specifically highlighting its implications for diagnosis and management.

## 1. Introduction

The ocular surface is constantly exposed to the environment, and a variety of bacteria inhabit it. These bacteria are known to be involved in maintaining homeostasis and contributing to various pathological conditions. The microbiota of the ocular surface is controlled by antimicrobial components such as IgA [1], lysozyme [1], lactoferrin [2], and defensins [3], which are secreted by lacrimal glands, accessory lacrimal glands, and conjunctival epithelium including goblet cells. Additionally, antimicrobial substances from meibum (the secretion from the meibomian glands) also play a role [4]. Therefore, not only the conjunctiva but also the tear film and surrounding eyelid margin, including the meibomian glands and eyelid skin, influence the composition of microbes on the ocular surface. Since the 19th century, numerous studies have been performed on the microbiota of the ocular surface using traditional bacterial cultures. Overall, these findings revealed that, in healthy subjects, the most commonly isolated aerobes are coagulase-negative staphylococci (CoNS), and the most commonly isolated anaerobes are *Cutibacterium acnes* (*C. acnes*), formerly *Propionibacterium acnes* (*P. acnes*) [5,6]. *Corynebacterium* spp. and *Staphylococcus aureus* (*S. aureus*) are less commonly isolated bacteria [5]. Recent 16S rRNA sequencing techniques have revealed that the microbiota of the conjunctiva and meibum in healthy adult subjects is highly diverse, with *C. acnes* being the most abundant; however, this diversity declines with aging [7].

*C. acnes* is a lipophilic Gram-positive bacillus which normally inhabits human sebaceous follicles [8]. *C. acnes* is considered an anaerobic aerotolerant as it possesses enzymes such as superoxide dismutase and peroxidase, to detoxify oxygen, allowing it to grow in the presence of oxygen [9]. It is well-known for its central role in the production of acne lesions in acne vulgaris [10]. In addition, *C. acnes* is implicated in a variety of clinical conditions, particularly those associated with medical devices and surgical procedures [11,12]. In the human eye, *C. acnes* has become known for its role as a causative bacterium in conditions such as late-onset endophthalmitis post-cataract surgery [13,14,15,16], infectious keratitis, particularly due to contact lens wear [17,18,19], meibomitis-related keratoconjunctivitis [20,21,22], chalazia [23], and granuloma formation in the retina of ocular sarcoidosis [24,25].

For a microbiological diagnosis, *C. acnes* is typically cultured on anaerobic blood agar [26], and it requires a minimum incubation period of 7 days [27]. In the field of ophthalmology, specimens obtained from sources such as corneal ulcers or vitreous fluid are usually very small, and the yield for detecting even obvious infections, such as infectious keratitis (52%) [28] and postoperative endophthalmitis (70%) [29], is relatively low. In contrast, polymerase chain reaction (PCR) is a more useful method for rapid and sensitive diagnosis compared to traditional culture techniques [30]. However, it has the disadvantage of a high rate of false positives due to commensal or dead bacteria [30]. Thus, the accurate identification of *C. acnes* is essential for proper diagnosis.

This review explores the dual role of *C. acnes* as both a commensal bacterium and a pathogen, acting as both an infectious agent and a trigger of inflammation in ocular diseases. By shedding light on the implications of *C. acnes* in the diagnosis and management of ocular inflammation, this review aims to enhance our understanding and lead to more targeted and effective therapeutic strategies.

## 2. *C. acnes* as a Commensal Bacterium on the Ocular Surface and Eyelid Margin

Numerous studies have investigated the bacteriology of the conjunctiva using traditional bacterial cultures. Anaerobes such as *C. acnes*, which typically appear in cultures after approximately 7 days [27], were initially overlooked by standard methods in previous years. In 1974, *C. acnes* was identified as a major component of normal skin and ocular flora [31]. It has been consistently recovered from the conjunctiva of healthy individuals [32] and the eyelid margin [33] along with aerobic bacteria such as *Staphylococcus* spp. and *Corynebacterium* spp. *C. acnes* is also found in meibum, albeit less frequently than coagulase-negative staphylococci (CoNS), which are predominant aerobes [33]. These findings have remained consistent in subsequent studies [5,34].

Since the early 21st century, microbiome research has advanced significantly with the advent of 16S rRNA gene sequencing. A recent study has shown that, in newborns, the conjunctival microbiome is primarily composed of Proteobacteria, followed by Actinobacteria and Firmicutes, with *C. acnes* being the most abundant bacterium [35]. An adult healthy conjunctiva is predominantly composed of Firmicutes, Actinobacteria, and Proteobacteria [36,37]. When compared to the microbiome of the eyelid skin, conjunctival sac, and meibum in the same eye in healthy young adults, both the conjunctival and meibum microbiomes exhibit a high alpha-diversity with *C. acnes* predominating [7]. In contrast, healthy elderly adults show a microbiome more similar to that of the eyelid skin, with approximately 30% displaying a low diversity of microbiomes dominated by *Corynebacterium* spp. or *Neisseriaceae* [7]. The microbiome of eyelid skin is relatively simple and typically dominated by *C. acnes* in the young but dominated by *Corynebacterium* spp. or *Neisseriaceae* in the elderly [7]. Although there are clear age-related differences, sex differences appear to be insignificant [7]. Several studies have highlighted microbial compositional changes in conditions such as dry eye and meibomian gland dysfunction (MGD) [38,39,40]. Recently, the microbiome associated with MGD has been reported to exhibit an increased abundance of lipophilic *Corynebacterium* spp., such as *C. macginleyi*, compared to normal subjects [41]. Additionally, *Pseudomonas* becomes more prevalent in cases of MGD accompanied by lacrimal dysfunction, which may contribute to the development of conjunctivitis [41]. These changes are often accompanied by a relative decline in the abundance of *C. acnes*, suggesting that *C. acnes* might play an important role in maintaining homeostasis on the ocular surface and eyelids.

## 3. *C. acnes* as an Infectious Pathogen: Postoperative Endophthalmitis and Keratitis

Although *C. acnes* is one of the most predominant commensal bacteria on the healthy ocular surface, it can be an infectious pathogen. The first case series of chronic endophthalmitis after extracapsular cataract extraction and intraocular lens (IOL) implantation caused by *C. acnes* was published in 1986 [13]. This condition is characterized by a white, intracapsular plaque composed of sequestered organisms in the relatively anaerobic peripheral capsular bag [16]. Since then, about 100 cases have been reported over eight case series [13,14,15,16,42]. The inflammation caused by *C. acnes* typically has a delayed onset compared to that caused by other common bacteria, such as CoNS [43]. This delay is due to *C. acnes*’s slow proliferation as an anaerobic microbe and its resistance to killing and degradation by neutrophils and monocytes [44]. Although the exact incidence of *C. acnes* endophthalmitis is not well-defined, chronic onset endophthalmitis following cataract surgery has been reported at a rate of 0.017% [45]. Diagnosis can be made through traditional anaerobic bacterial culture and/or PCR from aqueous and/or vitreous samples. Cultures take up to a week to yield results. In contrast, PCR offers advantages over bacterial cultures; for example, *C. acnes* was identified in 94% of vitreous samples by PCR compared to only 24% by culturing, whereas culturing identified *C. acnes* in 0% of aqueous samples [46]. Bacterial cultures obtained from undergone vitrectomy proved the presence of *C. acnes* in the vitreous and capsule and were shown to inhibit CD8+ T cells, which may play a role in the persistent inflammation in cases of *C. acnes* endophthalmitis [47]. The treatment is sometimes not successful because inflammation can recur many months and years after the initial treatment. The administration of topical and intraocular antimicrobial agents alone typically does not eradicate the organism because *C. acnes* can produce biofilms. These biofilms have been observed on the IOL haptics using scanning electron microscopy (SEM) [48] and can also become encapsulated in the peripheral capsule. Case reports of endophthalmitis due to *C. acnes* after an Nd: YAG capsulotomy [14,16] suggest the existence of *C. acnes* in the capsular bag with the IOL. Therefore, pars plana vitrectomy (PPV) and IOL extraction with antimicrobial treatment have been recognized as effective treatments [16,42].

*C. acnes* can also cause infectious keratitis. The first report was published in 1992 [17]. The characteristic findings are small ulcers with deep stromal infiltration evenly distributed in the cornea associated with anterior chamber cell reaction [17,18,19]. To identify *C. acnes*, an anaerobic bacterial culture is required with a minimum of 7 days of incubation. The risk factors for *C. acnes* keratitis (CAK) are considered to be similar to those of other infectious keratitis such as contact lens usage, ocular trauma, and ocular surgery. *C. acnes* is generally susceptible to most antimicrobial agents, including cephalosporins, fluoroquinolones, tetracyclines, erythromycin, vancomycin, and clindamycin [49]. However, recent findings indicate that it has developed varying degrees of resistance to clindamycin, macrolides, and tetracyclines [50]. For the topical treatment of CAK, fluoroquinolones are used extensively [51]. However, vancomycin is reportedly the most effective antimicrobial agent, as it has a lower minimum bactericidal concentration against *C. acnes* compared to other antimicrobial agents [19,52]. The visual prognosis is usually good in mild to moderate cases; however, it could be worse in severe cases [19].

## 4. *C. acnes* as a Trigger of Inflammation: Meibomitis-Related Keratoconjunctivitis (MRKC), Chalazion, and Ocular Sarcoidosis

Previous studies revealed that *C. acnes* is a potent inflammatory stimulus that can activate complement [53], produce serum-independent polymorphonuclear leukocyte (PMN) chemotactic factors [54], stimulate the release of lysosomal enzyme from human PMN [55], and selectively inhibit suppressor/cytotoxic T lymphocytes [56,57]. Furthermore, *C. acnes* has been used in animal models to induce granulomas in the lung, liver, and cornea [58,59,60]. Granuloma is a result of a chronic delayed-type hypersensitivity (DTH) response.

### 4.1. MRKC

*C. acnes* is a commensal bacterium in the meibomian glands [7,33,34]. They are modified sebaceous glands which produce and secrete lipids onto the eyelid margin, spreading onto the aqueous layer of tear film. It is now widely recognized that the condition of the meibomian gland has a direct impact on the ocular surface condition. Phlyctenular keratitis (PK), which is a disorder characterized by inflammatory corneal nodules predominantly in young females, has been reportedly recognized as phlyctenular-type of meibomitis-related keratoconjunctivitis (MRKC) since corneal phlyctenules (cellular infiltrates) are often located at the extension of meibomitis [20,21,22,61]. Bacterial culture of meibum from PK was highly positive for *C. acnes* compared with that from age-matched females [20,22]. Laboratory research has proven that *C. acnes* can induce DTH response in the rat cornea; heat-killed *C. acnes* can induce inflammatory cellular infiltration in the *C. acnes*-immunized rat cornea [60]. An immunohistochemical study revealed that CD4+ T cells infiltrated predominately over CD8+ T cells [60], a finding which is compatible with those of immunohistochemical studies of conjunctival phlyctenules [62]. Therefore, *C. acnes* can be causative for both meibomitis and corneal/conjunctival phlyctenules (cellular infiltrates) in MRKC. There seems to be a specific human leukocyte antigen (HLA), such as HLA-A26, B-35, or DRB1*0802, which is associated with *C. acnes* involvement [20]. Based on these findings, antimicrobial treatment specifically targeting *C. acnes* is essential and effective in curing phlyctenular-MRKC [20,21,22]. Phlyctenular-MRKC shares common clinical characteristics (female predominance, bilateral involvement, history of chalazia, and effectiveness of antimicrobial treatment) with childhood ocular rosacea, phlyctenular keratoconjunctivitis, and pediatric blepharokeratoconjunctivitis (PBKC) [20]. In 2024, an international consensus was reached on the definition and diagnostic criteria, widely defining these diseases as “PBKC” [63]. PBKC is often overlooked but is a sight-threatening inflammatory ocular surface disease, so it is important to consider *C. acnes* involvement when encountering corneal infiltrates with meibomitis.

### 4.2. Chalazion

Chalazion is a chronic inflammatory granuloma caused primarily by the obstruction of the meibomian gland orifices leading to the retention of meibum [64]. Histological examinations in the 19th century revealed its granulomatous nature, and its pathogenesis has been widely accepted as a foreign-body granuloma due to the degeneration products of meibum, i.e., “lipo-granuloma” [64]. Several pathogens had been proposed as risk factors for chalazion; however, no pathogenic bacteria had been proven to be causative until recently. Patients with phlyctenular-MRKC often have a history of chalazia [20,22]. An immunohistochemical examination using a *P. acnes*-specific monoclonal antibody (PAB antibody) [65] on chalazion tissues obtained from surgeries revealed positive staining for *C. acnes*, with these bacteria amid clusters of neutrophils as well as epithelioid macrophages and lymphocytes, i.e., “immune granuloma” [23]. This was the first report which implies the possible role of anaerobic *C. acnes* in granuloma formation in chalazion. The characteristic of *C. acnes* resistant to killing and degradation by human neutrophils and monocytes results in long-standing inflammation or granuloma development in the meibomian glands. For the treatment of chalazion, not only steroid usage to reduce the immune reaction but elimination of *C. acnes* with antimicrobial agents is effective.

### 4.3. Ocular Sarcoidosis

A preliminary case series study using PCR techniques on vitreous samples supports the involvement of *Propionibacterium* species in ocular sarcoidosis [66]. Later, two studies were conducted to explore *C. acnes* involvement in sarcoid granuloma in the retina [24,25]. The specimens obtained from vitrectomy were examined immunohistochemically with PAB antibody [24,25]. They contained the granulomas which were mainly observed in the inner retinal layer filled with CD4+ cells and CD68+ cells, indicating the Th1 immune response [25]. *C. acnes* has been isolated at a high ratio from the lymph nodes of the pulmonary hilum in the sarcoidosis patients compared to those from the patients with other disease, which leads *C. acnes* be recognized as the most implicated etiological bacterium for sarcoidosis in Japanese patients [67,68]. Obviously, *C acnes* is not commensal in the retina and the mechanism by which *C. acnes* reaches the retina is not clear. One possibility is that granulomas were caused by the hematogenous transmission of *C. acnes* as *C. acnes* was mainly detected in the granulomas in the inner retinal layer, where retinal vessels are mainly distributed [25].

## 5. Pathogenesis for Granuloma Formation for Chalazion and Sarcoidosis

Although *C. acnes* plays a significant role as a trigger for granuloma formation in both chalazion and sarcoid granuloma, the pathogenesis of these granulomas differs. Sarcoidosis is a systemic inflammatory disease characterized by the formation of noncaseating epithelioid granulomas in multiple organs, including the lungs and eyes [69]. Granuloma formation is a cellular attempt to contain an offending agent that is difficult to eradicate [70]. Foreign body granulomas are formed by agents with weak antigenicity, while epithelioid cell granulomas are formed by agents with strong antigenicity inducing Th1 immune response [71]. In patients with Th1 hypersensitivity to *C. acnes*, granulomatous inflammation is triggered by intracellular proliferation of the bacterium, resulting in granuloma formation [72]. In contrast, *C. acnes* does not appear to cause intracellular infection in chalazion, as clusters of *C. acnes* are surrounded by neutrophils and epithelioid cells [23]. This could explain the focal inflammation of the meibomian glands.

## 6. Conclusions

In summary, *C. acnes* is the most predominant anaerobic bacterium on the ocular surface and eyelid. As a commensal bacterium, it contributes to ocular surface homeostasis. However, it can also act as an infectious pathogen, causing late-onset postoperative endophthalmitis and keratitis, or as a trigger of immunoreactions resulting in long-lasting inflammation and granuloma formation on the cornea, meibomian glands, and retina. Managing inflammation and eliminating *C. acnes* is an effective treatment for curing infections and granulomas.
